# Physicochemical, antioxidant and sensory properties of Mango Sorbet containing L-theanine as a potential functional food product

**DOI:** 10.1007/s13197-022-05570-6

**Published:** 2022-08-16

**Authors:** Jackson Williams, Nathan M. D’Cunha, Jane Kellett, Ekavi N. Georgousopoulou, Andrew J. McKune, Duane D. Mellor, Nenad Naumovski

**Affiliations:** 1 Discipline of Nutrition and Dietetics, Faculty of Health, University of Canberra, Canberra, ACT 2601 Australia; 2 Functional Foods and Nutrition Research (FFNR) Laboratory, University of Canberra, Ngunnawal Country, Canberra, ACT 2617 Australia; 3 Australian National University Medical School, Australian National University, Canberra, ACT 2605 Australia; 4 School of Medicine, The University of Notre Dame, Sydney, 2010 Australia; 5 Centre for Health and Medical Research, ACT Health Directorate, Canberra, ACT 2601 Australia; 6 Faculty of Health, University of Canberra Research Institute for Sport and Exercise, Bruce, ACT 2617 Australia; 7 Discipline of Biokinetics, Exercise and Leisure Science, School of Health Sciences, University of KwaZulu-Natal, Durban, 4000 South Africa; 8 Aston Medical School, Aston University, Birmingham, B4 7ET UK; 9 Department of Nutrition-Dietetics, School of Health and Education, Harokopio University, 17671 Athens, Greece

**Keywords:** L-theanine, Antioxidant, Sensory evaluation, Dietary bioactive, Nutraceutical

## Abstract

The non-proteinous amino acid L-theanine (L-THE) is associated with a range of health benefits including improvements in immune function, cardiovascular outcomes and cognition. The aims of this study were to develop a food product (mango sorbet; ms-L-THE) containing physiologically relevant doses of L-THE (0.2/100 g w/w) and determine its antioxidant, physicochemical and sensory properties in comparison to a mango sorbet without L-THE (ms). Total phenolic and flavanol content, and antioxidant analysis (DPPH, FRAP and ABTS) were determined spectrophotometrically. Both products were also evaluated for acceptability and likeability in healthy participants using the 9-point hedonic scale. Any differences that could be caused by the addition of L-THE were examined using the triangle test. Results indicated no significant differences between ms-L-THE and ms in taste of the products (p > 0.05), and the ms-L-THE was well received and accepted as a potential commercial product. Findings of the DPPH assay indicated significant difference between the two products (p < 0.05). In conclusion, we have successfully created a mango sorbet that contains a potentially physiologically relevant concentration of L-THE with antioxidant properties that could be used as a novel method of L-THE delivery to clinical and healthy populations.

## Introduction

The worldwide consumption of Green Tea (GT) as an everyday, habitual or therapeutic beverage date back to the 10th century. Its potential beneficial health effects are reported to derive from several bioactive constituents that are associated with improvements in antioxidant responses (Li et al. 2012), cognitive function and numerous cardiovascular benefits (Juneja et al. 1999; Vuong et al. 2011; Williams et al. 2018). Additionally, the consumption of GT is associated with eliciting a relaxation response, which is predominately ascribed to the unique amino acid L-theanine (L-THE) (Juneja et al. 1999; Nobre et al. 2008). L-THE is a non-proteinogenic amino acid commonly found in GT tea leaves, which in its pure form of is described as a white crystalline powder with no odour and a slightly sweet taste that presents the unique *‘umami’* flavour sensation (Cooper 2012; Juneja et al. 1999; Narukawa et al. 2014).

The consumption of L-THE in humans is relatively safe, with administered doses of up to 900 mg/day exhibiting no apparent side-effects (Sarris et al. 2019). Paired with the recent commercial availability of the relatively pure form of L-THE and the growth in popularity in countries such as Japan and the United States, there is considerable potential for the development of functional food products containing quantifiable doses of this amino acid (Williams et al. 2016).

The term *‘functional food’* is commonly associated with health-enhancing food items designed to beneficially effect one or more functions in the body beyond the capabilities of the existing food by providing physiologically active compounds not ‘*naturally*’ found in them (Siró et al. 2008; Stanton et al. 2005). This relatively new concept of bioactive delivery provides an opportunity to rely less on the use of typical pharmaceutical drug delivery systems (i.e. capsules, tablets or syrups), and utilise the food matrix as a form of delivering bioactive ingredients at potentially therapeutic doses. Regardless of the method of delivery in which the bioactive is ingested, the functional food needs to be easily accessible and palatable. In this instance, the physiological responses ascribed from consuming pure L-THE occur at doses ranging from 0.05 to 0.4 g, equivalent to 2–15 cups of GT. This amount of tea is relatively difficult to achieve through ‘*normal*’ tea consumption in western society (on a daily basis). Therefore, the creation of a functional food product containing physiologically relevant quantities of L-THE is one method that can establish a relationship between food ingestion and the potential health benefits of pure L-THE (White et al. 2016).

The development of functional food poses several challenges such as the choice of ingredients and the method of delivery may influence health outcomes. For example, the use of macromolecules (proteins, fats), different stabilizers and flavour enhancers are commonly seen in several different food products. The reason for their use is predominately related to the improvements in the rheological, structural, and organoleptic properties. However, some of the food matrix components (in addition to the functional ingredient) can also be associated with the development of chronic diseases. For example, the excessive quantities and frequency of consumption of high sugar products are associated with the onset and development of several cardio-metabolic chronic diseases. Therefore, food product development should be precisely engineered to maintain the organoleptic properties, extension of shelf life and stability of the functional ingredient or additive while minimising the potentially negative health effects of other ingredients included in the formulation (Galanakis 2021; Hasler 2002).

To our knowledge, only two human trials have investigated the effects of L-THE incorporated into a functional food product. A relatively recent study provided a prospective outlook on the potential post-consumption attention enhancing effects of chocolate containing L-THE (128 mg) (Montopoli et al. 2015). In this study, it appears that there was an inhibitory neural sympathomimetic effect that acutely lowered blood pressure post consumption of the L-THE chocolate food product. In the other study, L-THE was provided in a supplemented beverage (200 mg) to 36 healthy adults, supporting the claims for potential *‘anti-stress effects’* of L-THE (White et al. 2016).

On a similar note, the antioxidant effect of L-THE can be potentially utilised to promote the health effects of functional food products. From a scientific perspective, antioxidant compounds have the ability to act as scavengers of reactive oxygen species (ROS) and potentially prevent ROS induced cellular damage (Naumovski et al. 2015). L-THE demonstrates antioxidant characteristics through the maintenance of intracellular glutathione levels (Sugiyama and Sadzuka 2004), as well as enhancing the antioxidant activity of hepatocytes that may potentially aid in the prevention of alcohol-related liver injury in vivo models (Li et al. 2012).

For foods to become commercially acceptable and in particular for functional food products to be effectively consumed over time, it is pivotal that food products are favoured by consumers. Several measures already exist that enable food manufacturers to determine whether a product will be liked, including numbered scales (hedonic scales, general likeability scales), and more recently, micro feedback visual analogue scales that involve faces attributed to how a product is perceived by the consumer (Wichchukit and O’Mahony 2015). Therefore, the aim of this study was to develop a mango sorbet that contains L-THE (ms-L-THE; 0.2/100 g w/w) and examine its physicochemical, antioxidant and organoleptic properties in comparison with a mango sorbet (ms) without L-THE.

## Materials and methods

### Materials and reagents

The mango fruit as a carrier for flavour of the sorbet was chosen due to the potentially low binding affinity with L-THE (less than 1%) through the analysis performed previously in our laboratory (data not published), and sorbet as the functional food base was selected due to the stability characteristics of L-THE (lower pH and temperature). Common mangos (Mango pieces, Coles Pty Ltd, Peru/Mexico), caster sugar (CSR, Yarraville, VIC, Australia), were purchased from local commercial suppliers. The L-THE (Suntheanine™, Taiyo Kagaku Co., Ltd, Japan) was purchased from Ingredient Resources Australia and New Zealand (Sydney, NSW, Australia), while the whey protein concentrate (WPC) was purchased from Professional Whey Pty/Ltd (Erina, NSW, Australia) and added to provide structural stability and rigidity to the food product.

All reagents; sodium carbonate, 2,2’-azino-bis sulfonic acid, aluminium chloride, catechin hydrate, DPPH, ethanol (> 98%) ferric chloride, Folin-Ciocaltu (FC) phenol reagent, gallic acid, hydrochloric acid (HCl), methanol (> 98%), potassium persulfate, sodium acetate trihydrate, sodium nitrite and 6-hydroxy-2,5,7,8-tetramethylchroman-2-carboxylic acid (Trolox) were all purchased from Sigma Aldrich (Castle Hill, NSW, Australia). Deionised water (DI Water) used was prepared on the day with resistivity greater than 18MΩcm^− 1^.

#### Preparation of food products

Both mango sorbets, ms-L-THE and the ms were prepared based on a previously published method (Naumovski et al. 2015) and adapted using mangoes and unflavoured whey protein powder to suit the purposes of this study. The mango pieces were pureed, caster sugar and WPC were added and mixed until dissolved completely and the resulting mixture was filtered to remove clumping. L-THE was added to one half of mixture and the resulting products were cooled (− 25 °C) in a commercially available ice cream maker (Breville Pty Ltd, Australia) Prepared sorbets (100 g) were stored in plastic containers at -18 °C until analysis and/or consumption.

### Macronutrient and Micronutrient Analysis

The Foodworks® (v8; Xyris Software, QLD, Australia) nutrient analysis software was used to calculate total macronutrient and some micronutrient profiles of the developed functional mango sorbets. In addition, total amino acid profiles were performed using the relevant quantities as provided by official nutritional panels of the relevant food products.

### Colorimetric analysis

Colour was measured using the commercially available Apple smartphone (iPhone 6, Apple Inc, Sydney, Australia) application Colour Detector (NanoSpark, Senasys, Altoona, Wisconsin USA). The smartphone was attached to a retort stand at specific height (35 cm) and controlled environmental light, followed by the analysis of five replicates (3 ± 0.5 g) of each ms-L-THE and ms products. The red, green and blue values, as well as the HUE, saturation and value (lightness), were also determined.

### pH and refractive index

The pH of ten samples (5 ± 0.5 g) of both sorbets (ms-L-THE and ms) were measured using pH700 meter (Eutech Instruments, ThermoFisher, Australia) after sorbets were thawed at room temperature. The refractive index and soluble solids were analysed on ten samples (1 ± 0.1 g) of both sorbets were analysed using the handheld electronic refractometer (Bellingham & Stanley, UK). The soluble solids results were expressed as °Brix.

### Melting rate

The melting rate in both sorbets was evaluated by removing the frozen products from circular containers (100 ± 1 g) and placing them on a wire rack (mesh size 1 cm^2^) on top of a funnel in a temperature-controlled room (22.5 ± 0.5 °C). Measurements of the dripped volume were recorded in triplicates at 5 min intervals up until 180 min. The drip volume (ml) was plotted against time (min), and melting rate was determined and expressed as ml/min (Muse and Hartel 2004).

### Moisture content

Nine samples of both ms-L-THE and ms (1 ± 0.4 g) were taken from the top, middle and bottom of its storage container and were sequentially centrifuged (Sigma 4-16KS, Sigma, Germany) (448 × *g*; 10 min; 25 °C) followed by a second centrifugation (17,500 × g; 25 °C). The supernatant was removed while the remaining solids were dried using the commercial food dehydrator (24 h; 75 °C). The total moisture content was determined using the gravimetric method (Official Methods of Analysis, 2016).

### Antioxidant and Phytochemical Content

All antioxidant assays were analysed in triplicates.

#### Methanolic extraction

Total phenolic, flavonoid and antioxidant capacity were determined in methanolic extracts of the mango sorbet following adapted methods (Thaipong et al. 2006). The extraction of antioxidants was performed by mixing the sorbet sample (1.12 ± 0.25 g) with methanol (70%) at a 1:4 ratio and sonicated in a water bath at maximum power for 15 min in 2 ml microcentrifuge tubes. The resulting mixtures were centrifuged (Sigma 4-16KS, Sigma, Germany) (14,000 × *g*; 15 min; 4 °C) and clear supernatant was used to determine the total flavonoid, phenolic and antioxidant analysis.

#### Total phenolic content

Total phenolic content (TPC) was performed based on adapted FC methods (Kähkönen et al. 1999). The absorbance readings were measured at 765 nm against the blank spectrophotometrically (Novaspec Plus, Unicam Sistemas Analíticos, Lisbon, Portugal) and values were represented as mg gallic acid equivalents (mg_GAE_).

#### Total flavonoid content

The total flavonoid content of both mango sorbet extracts was determined using an adapted method (Wu and Ng 2008). Absorbance was determined at 510 nm spectrophotometrically (Novaspec Plus, Unicam Sistemas Analíticos, Lisbon, Portugal). A serial dilution curve, using catechin hydrate as the standard were prepared and results were expressed as mg of catechin hydrate equivalents (mg_CHE_).

#### The 1,1-diphenyl-2picrylhydrazyl radical scavenging activity (DPPH) assay

The DPPH radical scavenging activity method was performed based on previously published methods (Thaipong et al. 2006). Absorbance readings were determined with methanol as the blank at 515 nm spectrophotometrically (Novaspec Plus, Unicam Sistemas Analíticos, Lisbon, Portugal). The absorbance values were expressed as µM Trolox equivalents per gram (µM_TE_) (Hipólito et al. 2016; Thaipong et al. 2006).

#### Ferric reducing antioxidant power assay (FRAP)

The FRAP method was based on the previously published method (Thaipong et al. 2006). The absorbance readings were taken at 593 nm spectrophotometrically (Novaspec Plus, Unicam Sistemas Analíticos, Lisbon, Portugal) against the working FRAP reagent as the blank. Results were expressed as µM Trolox equivalents per gram (µM_TE_) (Hipólito et al. 2016; Thaipong et al. 2006).

#### The 2,2’-azino-bis (3-ethylbenzothiazoline-6-sulphonic acid (ABTS) assay

The ABTS analysis was based on previously published methods (Thaipong et al. 2006). The absorbance was recorded against a methanol blank at 734 nm spectrophotometrically (Novaspec Plus, Unicam Sistemas Analíticos, Lisbon, Portugal). Results were expressed as µM Trolox equivalents per gram (µM_TE_) (Hipólito et al. 2016; Thaipong et al. 2006).

### Taste and product acceptability

#### Human Ethics approval

The approval to conduct this study was granted by the Human Research Ethics Committee of the University of  Canberra (HREC: 2016-16-42) and informed written consent was obtained from all the participants prior to commencement of each study.

#### Participants selection and exclusion Criteria Exclusion

For the sensory evaluation, 50 healthy untrained participants aged between 18 and 62 ± 10.26 years old (Males = 22; Females = 28), were recruited. Participants were excluded if they consumed any green tea, or green tea extracts, functional foods including cholesterol-lowering margarines, weight loss supplements or any commercial dietary products associated with weight loss. Participants were also excluded if they were suffering or had suffered from the any active pulmonary, hematologic, hepatic, gastrointestinal, renal, premalignant, malignant illnesses, diabetes (type I and type II) and have any thyroid dysfunction that may be exacerbated due to the consumption of the ingredients used in the preparation of the mango sorbet. In addition, females were excluded if they were pregnant.

#### Triangle test and sensory evaluation

The participants attended one clinic in total and were asked to provide sensory evaluation of the two sorbets using a triangle test and then rate the product for the categories of taste, texture, colour and overall using a 9 point hedonic scale adapted from the Society of Sensory Professionals (SOSP, 2019).

The likeability and acceptability of the foods products were determined using the scores on the hedonic scale, after the consumption of each 5 g food product (one for each sorbet). Participants were required to mark the scale according to likeability with 1 being extremely disliked to 9 being extremely liked.

For the purposes of the triangle test, procedures were followed as per the guidelines of the Society of Sensory Professionals (SOSP, 2016). Each participant consumed three sets of 5 g samples of sorbets in triplicates (total of 3 combinations of ms and ms-L-THE). All samples were provided in a randomised double-blind, placebo-controlled manner. Randomisation for both hedonic and triangle tests was performed *via* allocation of coded numbers using a randomising software (Randomness and Integrity Services; https://www.random.org/). The sequences were placed in a sealed envelope by an external person who did not participate in any components of the study.

### Data analysis

The Statistical Package for the Social Science (SPSS v23, IBM Statistics) and Microsoft Excel (v14.2.3) were used to analyse all data with alpha set at *p* < 0.05. The data for pH, refractive index, moisture content, colourimetry, phenolics, flavonoids and all antioxidant assays were compared using a paired two-tailed t-test (SPSS v23 IBM Statistics) and presented as mean with standard deviation. The triangle test was analysed using Chi-square analysis as per recommendations (SOSP, 2016) and presented as *X*^2^. The results of the hedonic scale responses were analysed using the Mann-Whitney U test to test the scores for likeability and acceptability scales, with each respective category ranked from 1 to 9 where a score of 1 indicated extremely disliked and 9 indicated extremely liked.

## Results and discussion

### Nutritional composition of Mango Sorbets

The total amino acid composition is presented in Table 1. The developed sorbets contained a total energy of 5.87 kJ/g with macronutrient distributions of carbohydrates (28.07 g; 26.1 g sugars), protein (8.33 g) and total fat (0.50 g) values per 100 g. Total micronutrients were determined for starches (1.53 g), vitamin C (> 5.36 mg), sodium (15.55 mg), potassium (> 0.28 mg), calcium (> 0.14 mg), niacin equivalents (> 1.39 mg), selenium (> 0.07 mg) and iodine (> 0.07 mg).

**Table 1:** The amino acid profile of the mango sorbet containing L-THE

Amino Acid	Quantity (g/100 g)	Amino Acid	Quantity (g/100 g)
Isoleucine (g)	0.58	Glycine	0.18
Leucine	1.00	Proline	0.54
Valine	0.29	Tyrosine	0.27
Lysine	0.87	Aspartic Acid	0.98
Methionine	0.20	Serine	0.48
Phenylalanine	0.30	Glutamic Acid	1.63
Threonine	0.66	Alanine	0.47
Tryptophan	0.18	Histidine	0.17
Arginine	0.25	L-Theanine	0.20^*^
Cysteine	0.23		

Both mango sorbets ms and ms-L-THE contain the same amounts of amino acids (*except L-THE which was only present in the ms-L-THE product)

The ms-L-THE has a relatively large total energy content (5.87 kJ/g), and a high amount of protein due to the addition of the WPC (8.33 g/100 g). It is worth noting that there are a variety of amino acids that have the potential to exhibit binding competitiveness against L-THE (methionine, leucine, isoleucine and valine), which are key amino acids that assist in muscle protein synthesis (Juneja et al. 1999; Zhang et al. 2017). Other amino acids such as alanine, serine, glycine, aspartic acid and glutamic acid show no significant changes in the presence of L-THE (Juneja et al. 1999) however, this mechanism of action was in animal models and may not necessarily translate to the human population.

### pH and °Brix

The pH levels were similar for both sorbets ms-L-THE (5.22 ± 0.02) and ms (5.23 ± 0.02) with no significant differences observed between the two products (*p* = 0.61). Similar findings were also observed for the soluble solid’s composition of the ms-L-THE (35.86 ± 0.21 °Brix) and ms (35.91 ± 0.47 °Brix) where addition of L-THE did not influence the soluble solid levels (*p* = 0.76).

The increase in pH which is a higher value than mangoes themselves (3.9–4.6) (USFDA, 2012) may likely contribute to the overall stability of L-THE which is quite stable in slightly acidic conditions of pH 5–6 (Williams et al. 2016) however, separate stability studies must be conducted in controlled environmental conditions to establish and further support this. In future, processes utilised to prepare foods containing L-THE or any functional food product should be carefully considered prior to development as processes that involve high temperatures and changes in pH could potentially alter the integrity and functionality of the food product.

### Melting rate of Mango Sorbet

**Fig. 1:**
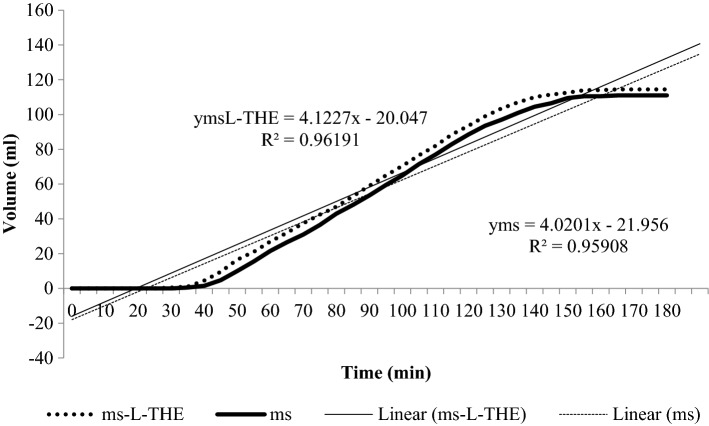
The melting rate of the mango sorbet without L-THE (ms) and the mango sorbet with the added 200 mg L-THE (ms-L-THE)

Both sorbets showed positive linear gradients (ms-L-THE 4.1227x and ms 4.0201x) before plateauing (Fig. 1). The average melting rates for ms-L-THE (0.62 ml/min) and ms (0.60 ml/min) were not significantly different (*p* = 0.81). It is known that the addition of sweeteners, can alter the melting rate of frozen food products where the addition of a 20 dextrose equivalent corn syrup decreased melting rates whereas the addition of a high fructose corn syrup sped up melting rates (Muse and Hartel 2004). Therefore, it is proposed that the high sugar content of this current food product may have accelerated the melting process, however in future studies, it may prove to be beneficial to formulate ms-L-THE with 20 dextrose equivalent corn syrup instead of pure sugar in order to decrease the melting rate (Muse and Hartel 2004).

### Moisture content

The total moisture content for ms-L-THE and ms are presented in Table 2. A total of 9 sorbet replicates of ms and ms-L-THE were measured and there were no significant differences observed between the samples (*p* = 0.43).

**Table 2:** Moisture content of the mango sorbet and mango sorbet with L-THE

Position within a sample	ms-L-THE (%)	ms (%)
Top	82.59 ± 0.01	80.48 ± 0.01
Middle	82.46 ± 0.02	82.06 ± 0.01
Bottom	81.74 ± 0.02	82.56 ± 0.00
Average	82.26 ± 0.02	81.70 ± 0.01

Values are represented as Mean±SD; *ms-L-THE*, mango sorbet with L-theanine; *ms*, mango sorbet without L-theanine

### Colourimetry

The colour quantification (Table 3) revealed no significant differences observed between the samples for Red (*p* = 0.19), Blue (*p* = 0.06) and for saturation (*p* = 0.13). Significant differences were observed for Green and HUE (*p* = 0.02 and *p* = 0.01 respectively). On visual appearance, the colour of both ms-L-THE and ms is similar to the colour amber, however an RGB colour chart indicates the colour of both L-THE and non-L-THE sorbets was closest to the colour ‘*goldenrod*’.

**Table 3:** Colorimetric values of the mango sorbet and mango sorbet with L-THE (Mean ± SD)

Sample	Red	Green	Blue	HUE	Saturation %	Value %
ms-L-THE	242 ± 2.55	206 ± 2.55*	26.4 ± 1.67	49.6 ± 0.55*	88.6 ± 0.01	95.8 ± 0.01
ms	238 ± 3.85	201 ± 1.10	30 ± 3.29	47.8 ± 0.40	87.2 ± 0.01	95.6 ± 0.01

*ms-L-THE*, mango sorbet with L-theanine; *ms*, mango sorbet without L-theanine

*Significant p < 0.05

### Total phenolic and total flavonoid content

The TPCs for ms-L-THE (37.48 ± 1.89 mg_GAE_) and ms (37.13 ± 4.38 mg_GAE_) were not significantly different (*p* = 0.93). Additionally, the average total flavonoid values for the ms-L-THE (7.05 ± 5.02 mg_CHE_) and ms (9.16 ± 1.57 mg_CHE_) were also not significantly different (*p* = 0.50).

Based on the small quantities of phenolics in this study, it is likely that the TPC would not pose any physiological relevance in future human trials but rather it could be used as a sensitive indicator of the food product quality. In addition to the TPC, minor quantities of flavonoid compounds were detected in both sorbets. There were differences in TPC and flavonoid content indicating that the L-THE did not bind to the polyphenolic or flavonoid structures of the mango protein or sugar matrixes. However, this is expressed with caution as the exact mechanism of conjugation between the L-THE and other polyphenolic compounds are still largely unexplored and combination with some ingredients (such as caffeine and some polyphenols) may affect the absorption of the L-THE in humans (Naumovski et al. 2015).

When compared with other studies the mean TPC for both the ms and ms-L-THE (37.31 ± 0.03 mg_GAE_) was similar to cantaloupe melon (38.5 mg_GAE_) and higher than galia melon (23.4 mg_GAE_) sorbets but lower than sorbet made from poppy (925.77–1,022.84 mg_GAE_) (Ekici 2014), encore mandarin (110 mg_GAE_), navelate orange (129.2 mg_GAE_) and a eureka lemon sorbet (69.7 mg_GAE_) (Hipólito et al. 2016). It is well established that the bioavailability and biological characteristics of polyphenols is affected when these compounds are consumed in synergy with other nutrients or food matrixes (Naumovski et al. 2015). The addition of WPC into the sorbet has the potential to form protein-polyphenol complexes, which have been shown to change the plasma kinetic profile of polyphenols that can affect overall absorption (Zhang et al. 2014). Studies have also reported that the high carbohydrate content in the present food product has the capacity to enhance polyphenol absorption, and therefore increase the maximal plasma concentration of polyphenols, as seen with fats (Zhang et al. 2014). However, further testing should be conducted in future studies of the current functional food product to confirm these findings.

### Antioxidant analysis

The values for DPPH, FRAP and ABTS are represented in Table 4. There were no significant differences between ms-L-THE and ms for FRAP (*p* = 0.95) and ABTS (*p* = 0.30). However, the DPPH values were significantly different (*p* = 0.01) between the two samples.

**Table 4:** Antioxidant characteristics of the mango sorbets

Antioxidant Assay	ms-L-THE (µM_TE_)	ms (µM_TE_)
DPPH	878.52 ± 41.80*	816.94 ± 21.51
FRAP	528.76 ± 49.45	526.82 ± 61.33
ABTS	448.93 ± 36.84	398.37 ± 76.90

Values are represented as Mean±SD; *ms-L-THE*, mango sorbet with L-theanine;*ms*, mango sorbet without L-theanine

*Significant p<0.05

These differences could be due to the use of the DPPH as this method is based on capturing the stable free radical due to the delocalization of spare electrons on the whole molecule (Brand-Williams et al. 1995), thus the higher the antioxidant characteristics of the sample, the higher the amount of DPPH radical absorbed (Hipólito et al. 2016). As such, DPPH does not dimerize, as occurs with other types of radicals. The delocalization on the DPPH molecule produces a purple colour when reacting with a hydrogen donor; the reduced form is generated accompanied by the disappearance of the purple colour (Thaipong et al. 2006). Furthermore, on reduction by an antioxidant species, the higher the antioxidant characteristics of the sample, the higher the amount of DPPH radical will be absorbed (Hipólito et al. 2016). In this instance, the assay was assumed to react to the antioxidant properties exhibited by the L-THE molecule within the ms-L-THE sorbet and can potentially represent the antioxidative action of the L-THE added to the product.

Although several in vitro and animal models have proposed health benefits and antioxidant activity of L-THE (Williams et al. 2016, 2018), the antioxidant effects of L-THE in foods are still relatively limited and unexplored. To the best of our knowledge, only two studies have reported the antioxidant activity of food products after the addition of L-THE (Kim et al. 2021; Xue et al. 2022). In a study by Kim et al. (2021), L-THE (extract) was used for the production of green tea jellies (a traditional dessert in several Asian cultures) using different types of jellying agents. The findings have indicated that combination of L-THE (35% extract) and several gums (tamarind:xanthan:locust bean = 2:3:5 (w/w/w)) has exhibited the most optimal textural properties and high DPPH scavenging activity. Unfortunately, the authors did not provide any detailed potential mechanisms of action that show how the addition of L-THE may exhibit antioxidant activity. Furthermore, a study by Xue et al. (2022) investigated the use of L-THE on improvements in emulsification stability and antioxidant capacity of diacylglycerol (commonly used as a fat substitute). The findings of this study proposed that L-THE can bind to the β-lactoglobulin (globular protein and major whey protein in milk). This complex was found to improve antioxidant capacity (measured by DPPH and ABTS) of diacylglycerol in addition to improvements of emulsification stability and droplet size distribution. The potential reasons for this could be due to the L-THE molecule containing hydrophilic and hydrophobic groups and theoretically provide acetyl-containing structure. The new structure can stabilize the emulsions however, the change in antioxidant activity after the application of L-THE/β-lactoglobulin still remains unexplained.

The FRAP method is based on the reduction of the Fe(III)-complex of TPTZ by antioxidants producing a Fe(II)-TPTZ chelate complex (Benzie and Strain 1996; Lafarga et al. 2018; Özyürek et al. 2011; Thaipong et al. 2006). However, since L-THE has no known properties resembling ferric molecules, the reaction did not show any interaction with the ms-LTHE sorbet. On a similar note, the ABTS decolourisation assay can also be used to determine the antioxidant capacity of both hydrophilic and lipophilic food antioxidants in biological compounds and food samples (Arnao et al. 2001; Re et al. 1999). Although, the ms-L-THE results did not indicate any significant differences in the antioxidant properties of the lipophilic or hydrophilic antioxidants. As these techniques have all shown differences in controlled laboratory settings, when analysing food products, it is best to apply a battery of different techniques to determine the total antioxidant content (Thaipong et al. 2006). Contextually, this could also indicate a relative stability and non-binding of L-THE to the food matrix. However, to measure this, extraction studies must be used to determine the total amounts of L-THE present in the non-bound form within food matrixes.

### Sensory evaluation

#### Triangle Test and Acceptance

In total 147 out of 150 triangle tests, and 50 hedonic scales were completed. Three of the results were excluded due to participant error in marking of one or more of the provided categories. The results have indicated no significant differences between the two sorbet products after the Chi-square analysis *X*^2^ (1, *N* = 50) = 3.84, (p < 0.05).

**Fig. 2:**
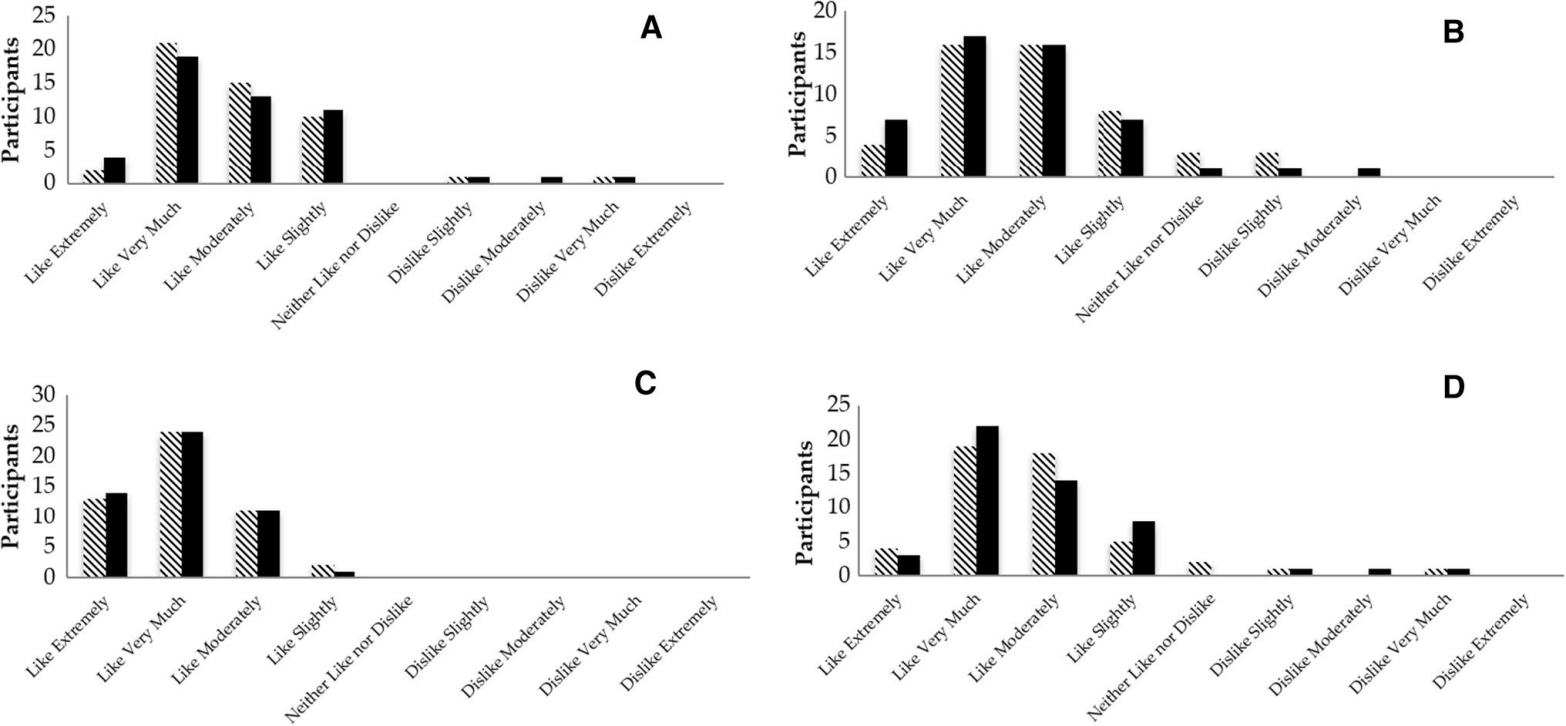
The results of the 9 point hedonic scale of the 50 included participants for the categories: Taste **a**, Texture **b**, Colour **c** and Overall likeability **d** for both ms-L-THE (mango sorbet with L-theanine) and ms (mango sorbet without the added L-theanine)

The 9-point hedonic scale likeability scores of ms-L-THE and ms (Fig. 2) indicates that data was not normally distributed and are displayed as median (1st and 3rd interquartile range). Both ms and ms-L-THE products were *‘liked moderately’* for *‘taste’* (Fig. 2 a) 7 (7, 8) and 7 (6, 8) (*p* = 0.89), respectively. For texture, both ms and ms-L-THE were *‘liked moderately’* (Fig. 2b) 7 (6, 8) and 7.5 (7, 8) (*p* = 0.20). The *‘colour’* of both ms-L-THE and ms was *‘moderately liked’* for ms and *‘liked very much’* for ms-L-THE (Fig. 2c) 8 (7, 9) and 8 (8, 9) (*p* = 0.44), respectively. The *‘overall’* likeability scores indicate that both sorbets were *‘liked moderately’* (Fig. 2d) 7 (7, 8) and 8 (7, 8) (*p* = 0.87) for ms and ms-L-THE, respectively.

The results for overall likeability analysis (Fig. 2) indicated that the highest frequency of reported responses fell in the ‘*moderately liked*’ category for both ms and ms-L-THE in categories taste, texture and overall, with the product being ‘*liked very much’* and *‘moderately liked’* for colour category for the ms-L-THE and ms, respectively. There were no significant differences in distribution in each respective category, which indicated that the product was widely accepted amongst the participants (Wichchukit and O’Mahony 2015).

The product differentiation amongst consumers can be determined using the several discriminatory tests that already exist specifically designed to determine differences between food products such as the *‘Triangle Test’*, *‘duo-trio’* and the *‘same-different’* tests (Rousseau et al. 1998). These tests are essential for different product development phases where may be a need for flavour matching (i.e. product blinding for clinical trials), and brand-related flavour matching. In most instances, the use of triangle tests to evaluate chilled food products may also present potential problems due to the time-temperature constraints required for product consumption (Rousseau et al. 1998). The difference in temperature has the potential to change the overall flavour and texture response due to taste receptors on the tongue, in particular transient receptor potential cation channel subfamily 5, which enhance gustatory nerve responses to sweet compounds when exposed to different temperatures where cold foods decrease sensory stimulation (Talavera et al. 2005). It is suggested that a change in temperature may affect product sweetness (where sweetness sensitivity increases linearly with temperature) related to sample perception. Indeed, temperature differences between the first and final blocks of randomly allocated frozen ms and ms-L-THE products may have been notably different due to the time the sample spent exposed to out of freezer conditions, however this did not affect the current study. This was accounted for in our study by providing both food products to participants within a set time frame based on melting rates whereby the product was consumed within 35 min of removal from storage.

## Conclusion

In conclusion, we have successfully developed a potential functional food product in the form of mango sorbet containing 0.2 mg/100 g of L-THE. The mango sorbet base (ms) used for the development of the potential functional food product (ms-L-THE) contain both moderate levels of phenolic compounds and minimal flavonoid levels. Furthermore, the ms-L-THE displayed its antioxidant activity but only in using the DPPH measurements, potentially through the hydrophobic binding to β-lactoglobulin. Furthermore, the results of the 9-point hedonic scale data highlight that the functional food product is *‘liked moderately’* by consumers for taste, texture, colour and overall categories. As a result, the designed ms-L-THE food product has the potential to be used a medium in delivering physiologically relevant L-THE doses in a variety of clinical and commercial settings.

## Data Availability

All data generated or analysed during this study are included in this published article.

## Abbreviations

L-THE: L-theanine
ms: mango sorbet
ms-L-THE: mango sorbet containing L-theanine
w/w: weight per weight
DPPH: 1,1-diphenyl-2picrylhydrazyl
FRAP: Ferric reducing antioxidant power
ABTS: 2,2’-azino-bis (3-ethylbenzothiazoline-6-sulphonic acid.
GT: Green tea
ROS: Reactive oxygen species
FC: Folin-Ciocaltu phenol reagent
TPC: Total phenolic content
mg _CHE_: Milligrams of catechin hydrate equivalent
HREC: Human research ethics committee
SOSP: Society of sensory professionals
USFDA: United states food and drugs administration
GAE: Gallic acid equivalents
